# Comparison of a guaiac and an immunochemical faecal occult blood test for the detection of colonic lesions according to lesion type and location

**DOI:** 10.1038/sj.bjc.6604996

**Published:** 2009-03-31

**Authors:** L Guittet, V Bouvier, N Mariotte, J P Vallee, R Levillain, J Tichet, G Launoy

**Affiliations:** 1 Cancers and Populations, ERI3 INSERM, UFR de Médecine, CHU de Caen, Avenue de la Côte de Nacre, F-14000 Caen, France; 2 Association Mathilde, 28 rue Bailey, F-14000 Caen, France; 3 Institut inter-Régional pour la Santé, 45 rue Parmentière, BP 22, F-37521 La Riche, Cedex, France

**Keywords:** colorectal neoplasms, faeces/chemistry, guaiac, mass screening, occult blood, sensitivity and specificity

## Abstract

We investigated variations in sensitivity of an immunochemical (I-FOBT) and a guaiac (G-FOBT) faecal occult blood test according to type and location of lesions in an average-risk 50- to 74-year-old population. Screening for colorectal cancer by both non-rehydrated Haemoccult II G-FOBT and Magstream I-FOBT was proposed to a sample of 20 322 subjects. Of the 1615 subjects with at least one positive test, colonoscopy results were available for 1277. A total of 43 invasive cancers and 270 high-risk adenomas were detected. The gain in sensitivity associated with the I-FOBT was calculated using the ratio of sensitivities (RSN) according to type and location of lesions, and amount of bleeding. The gain in sensitivity by using I-FOBT increased from invasive cancers (RSN=1.48 (1.16–4.59)) to high-risk adenomas (RSN=3.32 (2.70–4.07)), and was inversely related to the amount of bleeding. Among cancers, the gain in sensitivity was confined to rectal cancer (RSN=2.09 (1.36–3.20)) and concerned good prognosis cancers, because they involve less bleeding. Among high-risk adenomas, the gain in sensitivity was similar whatever the location. This study suggests that the gain in sensitivity by using an I-FOBT instead of a G-FOBT greatly depends on the location of lesions and the amount of bleeding. Concerning cancer, the gain seems to be confined to rectal cancer.

It is now established that screening by measuring faecal occult bleeding in average-risk populations can detect asymptomatic colorectal cancers and precancerous lesions (high-risk adenomas). A number of recently reviewed randomised trials have established the efficacy of average-risk population screening using the Haemoccult II guaiac faecal occult blood test (G-FOBT) to reduce specific mortality related to colorectal cancer (Hewitson *et al*, 2008). However, the sensitivity of this test for the detection of invasive cancer and adenomas limits the expected gain in terms of mortality. Over the past 20 years, an alternative type of FOBT has been developed based on the immunological detection of human haemoglobin (Hb), and offering greater sensitivity for the detection of invasive cancers and pre-neoplastic lesions (Morikawa *et al*, 2005; Castiglione *et al*, 2007; Guittet *et al*, 2007; Levi *et al*, 2007). With these tests, the gain in sensitivity appears to be higher for adenomas than for invasive cancers (Guittet *et al*, 2007). Several authors have concluded that bleeding from high-risk adenomas, measured by an immunochemical FOBT (I-FOBT), is lower than bleeding from invasive cancers (Ciatto *et al*, 2007; Levi *et al*, 2007). The influence of the location of the colonic lesion (in particular adenomas) on the amount of measured bleeding has been inconsistently observed (Ciatto *et al*, 2007; Levi *et al*, 2007). Because lesion detection is based on bleeding, the amount of bleeding is consequently likely to determine the detectability of the lesion. Our aim was thus to compare the sensitivity of the Magstream I-FOBT and the Haemoccult II G-FOBT according to the type and the location of lesions in an average-risk 50- to 74-year-old population.

## Methods

### Study design

As from June 2004, a screening programme has been implemented using a conventional G-FOBT, the Haemoccult II, for individuals aged 50–74 years in the geographic area of Calvados (Normandy, France). The first 30 000 attendants to screening were offered the possibility to join a study comparing an I-FOBT (Immudia/RPHA) and the conventional G-FOBT. This analysis focuses on the 20 322 subjects having performed both tests from 1 June 2004 to 31 December 2005.

The design of the study has been detailed in a previous publication (Guittet *et al*, 2007). Participants were asked to obtain, at home, two faecal samples on two different days for the I-FOBT and two faecal samples each from three consecutive stools for the conventional G-FOBT. The use of the same stools for I-FOBT and G-FOBT was not mandatory. No specific dietary restriction was stipulated. Samples of both tests were sent to the central analysis centre (Institut inter-Régional pour la Santé, Tours, France) where all tests were processed independently according to manufacturers’ recommendations, readers of the G-FOBT being blinded to the I-FOBT result. Guaiac faecal occult blood tests with at least one positive oval out of six were considered positive. The I-FOBT was processed using a Magstream 1000 automated device. The I-FOBT was considered positive when at least one of the two samples contained at least 20 ng ml^−1^ of Hb in the buffer, which corresponds to 0.1–0.2 mg Hb per gram stool. The subject and practitioner were informed of the overall screening procedure result, but were blinded to each individual test result. In the case of a positive result (i.e. at least one of the FOBT result was positive), the subject was invited to undergo a colonoscopy. The colonoscopy was performed blinded to the I-FOBT and G-FOBT results. The study was approved by the local ethics committee (Comité Consultatif de Protection des Personnes dans la Recherche Biomédicale) and all participants gave written informed consent.

Subjects were excluded from this analysis if the endoscopic examination was incomplete (caecum not visualised). However, if the colonoscopy was incomplete because of obstructing tumours or if the incomplete colonoscopy had been completed by another colonic examination (double-contrast barium enema or virtual colonoscopy) that failed to reveal any polypoid lesion, the results were included in the analysis. Hyperplastic polyps were not included as neoplasia. Advanced colonic neoplasia was defined as high-risk adenoma (adenomas measuring 10 mm or more, adenomas with high-grade dysplasia) or invasive cancer. Intramucosal carcinoma and carcinoma *in situ* were classified as adenoma with high-grade dysplasia. The criterion for diagnosing cancer was an invasion of malignant cells beyond the muscularis mucosa. If a subject had more than one polyp, the most advanced pathological lesion or the largest lesion was included in the analysis.

### Study population – Statistical analysis

The population of eligible subjects comprised of all subjects with both I-FOBT and G-FOBT analysable results who had also given informed consent, from 1 June 2004 to 31 December 2005 (*n*=20 322). Among the 1615 subjects with a positive screening test (positive G-FOBT and/or I-FOBT), 1387 (85.9%) performed the indicated colonoscopy. The frequency of subjects undergoing endoscopic examination did not differ depending on which FOBT was positive (G-FOBT and I-FOBT positive, 82.6%; G-FOBT positive only, 84.9%; I-FOBT positive only, 84.6%, *P*=0.77). Fifty-nine subjects with incomplete colonoscopy, and 27 for whom information on the polypoid lesion was insufficient, were excluded from the analysis. Consequently, 1277 subjects with at least one positive test and having completed a satisfactory colonoscopy were included in the analysis.

Because the confirmatory procedure (colonoscopy) was restricted to subjects classified as positive by at least one of the tests, the sensitivity and specificity of each test could not be directly estimated. We therefore compared the sensitivity of each test to detect high-risk adenomas or invasive cancers according to the location of lesions using the ratio of sensitivities (RSN) (Schatzkin *et al*, 1987; Cheng and Macaluso, 1997). Briefly, if we denote by *m*′_1_ the number of true positive subjects for the I-FOBT and by *n*′_1_ the number of true positive subjects for the G-FOBT, RSN is calculated as: RSN_I−FOBT/G−FOBT_=*m*′_1_/*n′*_1_. We compared the relative gain in sensitivity between lesions using a marginal regression approach involving a log-link function and an independence working covariance matrix by applying a generalised estimating equation (Sullivan Pepe and Alonzo, 2001).

The maximal faecal occult bleeding measured by the Magstream I-FOBT was compared according to location and type of lesions among subjects with a maximum of one lesion.

Statistical analysis was performed using SAS software version 9.1 (SAS Institute).

## Results

Table 1 shows the colonoscopy findings for the 1277 participants with at least one positive test and a satisfactory colonoscopy result. Among them, 390 (30.5%) had a positive G-FOBT and 1028 (80.5%) had a positive I-FOBT. Invasive cancers were detected for 43 subjects and at least one high-risk adenoma (size ⩾10 mm or high-grade dysplasia) was observed for 270 subjects without cancer. Table 2 illustrates that most of the high-risk adenomas were detected in the distal colon, whereas half of the invasive cancers were located in the rectum. Fourteen (34.1%) of the invasive cancers were detected at early stage of extension (stage T1-T2 N0 M0 of the Tumour Node Metastasis classification).

### Comparison of performance between G-FOBT and I-FOBT

Table 3 shows the results of colonoscopy according to the results of both tests: guaiac and immunochemical tests positive (G^+^I^+^), guaiac test positive and immunochemical test negative (G^+^I^−^), guaiac test negative and immunochemical test positive (G^−^I^+^). The positive predictive value (PPV) was lower for I-FOBT than for G-FOBT with regard to invasive cancers (4.0 *vs* 6.9%; *P*=0.03), and higher with regard to high-risk adenomas (24.3 *vs* 19.5%). Using the RSN, Table 3 compares the sensitivity of I-FOBT and G-FOBT for the detection of invasive cancers and high-risk adenomas according to the location of the lesion. The sensitivity of I-FOBT was higher than that of G-FOBT for invasive cancer (RSN=1.48 (1.16–1.89)) and high-risk adenomas (RSN=3.32 (2.70–4.07)). The increase in sensitivity for the detection of high-risk adenomas was significantly greater than that of invasive cancers (*P*<10^−3^). Concerning cancer, the increase in sensitivity of I-FOBT compared to G-FOBT was far higher for rectal cancers than for other localisations. In fact, the gain in sensitivity for cancer was restricted to invasive cancers located in the rectum (RSN_rectal cancer_>RSN_colonic cancer_, *P*=0.013), the gain for other localisations being non-significant. Table 4 illustrates that the majority of the invasive cancers missed by the G-FOBT and detected by the I-FOBT were good prognosis cancers (T1-T2 N0 M0) located in the rectum. Concerning high-risk adenoma, the gain in sensitivity did not depend on the location.

### Physiological rationale

#### Amount of bleeding according to the type and location of the lesion

**Figure 1:**
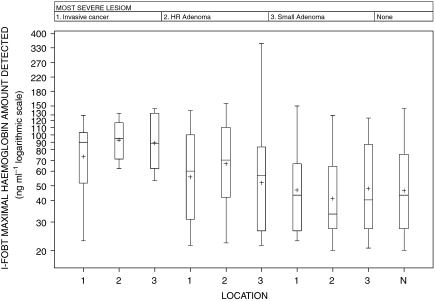
Distribution of the maximal haemoglobin amount measured in positive I-FOBT (immunochemical faecal occult blood test) subjects according to the most severe lesion and its location (subjects with only one lesion detected). HR adenoma, high-risk adenoma (adenoma ⩾10 mm or high-grade dysplasia). Location: 1. rectum+recto-sigmoidal junction; 2. distal colon; 3. proximal colon.

Figure 1 shows the percentile of distribution of the maximal amount of bleeding measured for each I-FOBT-positive subjects by the automatic I-FOBT reader according to the type and location of the lesion. Subjects with more than one lesion were excluded from this analysis. Independent of the location, the amount of bleeding of invasive cancers (mean=86.6 ng Hb per ml) was higher than that of high-risk adenomas (mean=71.9 ng Hb per ml) that was in turn higher than those of small adenomas (mean=52.7 ng Hb per ml) or normal colon (mean=54.3 ng Hb per ml). Bleeding amounts for cancer of the rectum were lower than those for distal or proximal colon cancer, although non-significant. Bleeding amounts for rectal cancers were only slightly higher than that of high-risk adenomas located in the rectum and distal colon. Bleeding amounts for high-risk adenomas of the proximal colon were similar to those for small adenoma or normal colon. In summary, severe lesions with the lowest amount of bleeding (high-risk adenomas and rectal cancers) correspond to those for which I-FOBT provides the most important gain in sensitivity.

#### Haemoglobin detection level using G-FOBT

**Figure 2:**
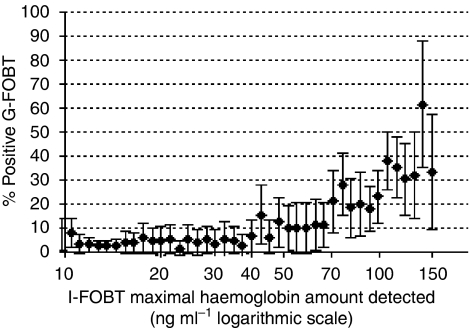
Frequency of positive G-FOBT (guaiac faecal occult blood test) according to result of the I-FOBT (immunochemical faecal occult blood test; two samples).

Figure 2 illustrates the variation in the G-FOBT positivity rate according to the amount of bleeding measured by the automatic I-FOBT reader. To the left of the figure (bleeding amount <40 ng Hb per ml), the rate of positive G-FOBTs remained around 2%, suggesting that G-FOBT is not capable of detecting bleeding less than 40 ng Hb per ml. To the right of the figure (bleeding amount ⩾40 ng Hb per ml), no gap in the positivity rate was observed, but rather an exponential increase in G-FOBT positivity with increased Hb content detected by the I-FOBT.

## Discussion

The extent of gain in sensitivity in screening for colorectal cancer when using I-FOBT instead of G-FOBT depends on the type and the location of lesions. It was higher for high-risk adenomas than for cancers. Among cancers, the gain in sensitivity was essentially confined to rectal cancers and concerned good prognosis cancers. This gain in sensitivity was related to the small amount of bleeding observed in such lesions.

Our study has several drawbacks. First of all, because subjects for whom both tests were negative underwent no further evaluation by colonoscopy, we were unable to provide an estimation of sensitivity and specificity for each test. However, the use of RSN allowed us to quantify the potential gain obtained through the substitution of G-FOBT by I-FOBT for each lesion type and location. Moreover, despite our large sample size, the number of discordant pairs was low. Therefore, the size of confidence intervals was probably underestimated (Cheng *et al*, 2000), and the effect of the location of invasive cancers on RSN may be lesser than it appears in Table 3. However, physiological rationale for these differences reinforces our conclusions. Furthermore, the measurement of bleeding amounts was provided by the I-FOBT itself. It therefore depended on the technical characteristics of this I-FOBT and could by biased. Only one I-FOBT was used, and any extrapolation of our results to other I-FOBTs should be performed with caution. Finally, a proportion of the subjects included in our study did not undergo colonoscopy. This is unlikely to have differentially biased the results because the proportion was similar whatever the test results, and colonoscopy was performed blinded to both.

Our study also has strengths. First, the study included asymptomatic average-risk subjects. This is of particular importance because the differential amount of bleeding between symptomatic (rectal haemorrhage) and asymptomatic cancers is higher for rectal than for colonic cancers (Macrae and St John, 1987). Moreover, the comparison (both globally and according to location) of sensitivity for the detection of invasive cancers and high-risk adenomas benefited from the paired comparison of the tests used in this study. Indeed, the sensitivity for the detection of invasive cancers for each test has been studied essentially using (with the exception of the Morikawa study) data from registries (detection or incidence methods (Day, 1985)) (Launoy *et al*, 1997; Morikawa *et al*, 2005; Castiglione *et al*, 2007). However, these methods cannot be used to detect high-risk adenomas because of their small probability of becoming symptomatic within 2 years, unless they evolve towards invasive cancer. Therefore, appropriate data are lacking to evaluate sensitivity for the detection of high-risk adenomas.

We observed a higher gain in sensitivity using I-FOBT rather than G-FOBT, for the detection of rectal rather than colonic cancers. Our results are consistent with previous studies, suggesting that the sensitivity of Haemoccult G-FOBT is stable whatever the location (Launoy *et al*, 1997) and that the sensitivity of Magstream I-FOBT is higher for rectal+distal colonic tumours than for proximal colonic tumours (Morikawa *et al*, 2005). A non-significant increase in the sensitivity of the OC-sensor I-FOBT was also observed for the detection of invasive cancers of the rectum *versus* the colon (Castiglione *et al*, 2007).

Several considerations require to be developed to explain the difference in sensitivity. They concern, first, the amount of bleeding and second, test processing.

In accordance with our results, several authors have observed that the sensitivity of the G-FOBT decreases as the amount of bleeding of the lesion increases (Morris *et al*, 1976; Stroehlein *et al*, 1976; Herzog *et al*, 1982; Ahlquist *et al*, 1985; Macrae and St John, 1987). As in other studies, in our population, the measured intensity of bleeding of invasive cancers was higher than that of high-risk adenomas (Levi *et al*, 2007; Ciatto *et al*, 2007). Moreover, we observed lower bleeding intensity in rectal cancers than in cancers in others locations, which is also in line with observations of Ciatto *et al* (2007) who found smaller although non-significant bleeding amounts for cancers of the rectum+distal colon rather than proximal colon.

Difference in sensitivity according to location can also be explained by test process considerations. The G-FOBT relies on the detection of peroxidase-like activity of haem in Hb; thus, the test detects both Hb and haem, which results either from the deterioration of Hb or from feeding. Both haem and Hb are deteriorated during intestinal transit and deteriorated globin and haem-derived porphyrins are not detected by Haemoccult (Goldschmiedt *et al*, 1988; Young *et al*, 1990). Herzog *et al* (1982) observed that the positivity of the G-FOBT was determined by the daily faecal blood loss and the anatomic location of colonic bleeding sites. They found that the G-FOBT was more frequently positive for distal rather than proximal colonic polyps of similar Hb content.

The Magstream I-FOBT relies on an antibody directed against the globin moiety of human Hb. Deteriorated Hb is not detected. Immunochemical faecal occult blood test may underestimate the intensity of bleeding in proximal polypoid lesions and, therefore, be less sensitive to such lesions. This could explain why Morikawa *et al* (2005) found that the sensitivity of the one-time Magstream for the detection of adenomas measuring over 10 mm was smaller for proximal than for distal lesions (11 *vs* 25%), whereas Herzog *et al* (1982) found no difference in bleeding intensity according to the location of the lesion. Consequently, the constant RSN we observed for the detection of high-risk adenomas throughout the low digestive tract, which could correspond to a similar variation in sensitivity of the I-FOBT and G-FOBT, is in accordance with these findings.

Our previous studies have demonstrated that I-FOBT is preferable to G-FOBT from a public health point of view because the availability of quantitative Hb measurement offers the possibility to choose a positivity rate offering both high sensitivity and high specificity (Launoy *et al*, 2005; Guittet *et al*, 2007). This study suggests that I-FOBT is also preferable to G-FOBT from a clinical point of view, because it is capable of detecting good prognosis cancers of the rectum and high-risk adenomas that are missed by G-FOBT. This is of particular importance because, in colorectal cancers, the proportion of those located in the rectum varies from 30 to 45% in different cancer registries (Parkin *et al*, 2005). The higher gain in sensitivity for good prognosis cancers should enhance the expected gain in survival through screening by using the I-FOBT. Further studies are needed to evaluate the relevance of adapting the confirmatory test to the level of positivity offered by the I-FOBT, using a rectosigmoidoscopy for subjects with a weak positive I-FOBT (small amounts of bleeding) and a colonoscopy for subjects with a strong positive I-FOBT.

## Figures and Tables

**Table 1:** Characteristics of subjects with positive tests and colonoscopy findings according to the kind of test (I-FOBT *vs* G-FOBT^a^)

	**Positive G-FOBT^b^ (*n*=390)**		**Positive I-FOBT^c^ (*n*=1028)**		**Overall (*n*=1277)**	
*Sex (%)*						
Male	176	(45.1)	553	(53.8)	649	(50.8)
Female	214	(54.9)	475	(46.2)	628	(49.2)
						
*Age*						
Mean (s.d.) (years)	63.65	(6.57)	63.53	(6.90)	63.44	(6.82)
50–54 (*n* (%))	41	(10.5)	133	(12.9)	162	(12.7)
55–59 (*n* (%))	82	(21.0)	228	(22.2)	286	(22.4)
60–64 (*n* (%))	85	(21.8)	202	(19.7)	257	(20.1)
65–69 (*n* (%))	98	(25.1)	237	(23.1)	299	(23.4)
70–74 (*n* (%))	84	(21.5)	228	(22.2)	273	(21.4)
						
*Colonoscopy findings*						
Invasive cancer	27	(6.9)	41	(4.0)	43	(3.4)
High-risk adenoma^c^	76	(19.5)	250	(24.3)	270	(21.1)
Small adenoma	57	(14.6)	211	(20.5)	251	(19.7)
Normal colon	230	(59.0)	526	(51.2)	713	(55.8)

a G-FOBT=guaiac faecal occult blood test; I-FOBT=immunochemical faecal occult blood test.

b Including 141 subjects who were positive for G-FOBT and I-FOBT.

c Adenoma ⩾10 mm or high-grade dysplasia.

**Table 2:** Characteristics of the subjects, location and extension of lesion according to colonoscopy findings

	**Invasive cancer (*n*=43)**		**High-risk adenoma (*n*=270)**		**Other (*n*=964)**	
*Sex (%)*						
Male	23	(53.5)	170	(63.0)	456	(47.3)
Female	20	(46.5)	100	(37.0)	508	(52.7)
						
*Age*						
Mean (s.d.) (years)	65.52	(6.18)	64.34	(6.68)	63.15	(6.88)
50–54 (*n* (%))	3	(7.0)	23	(8.5)	136	(14.1)
55–59 (*n* (%))	6	(14.0)	58	(21.5)	222	(23.0)
60–64 (*n* (%))	10	(23.3)	53	(19.6)	194	(20.1)
65–69 (*n* (%))	10	(23.3)	74	(27.4)	215	(22.3)
70–74 (*n* (%))	14	(33.6)	62	(23.0)	197	(20.4)
						
*Location* a						
Rectum^b^	23	(52.3)	55	(19.4)	NA	
Distal colon^c^	15	(34.1)	177	(62.5)	NA	
Proximal colon^d^	6	(13.6)	51	(18.0)	NA	
Missing	0		5		NA	
						
*Stage* a,e						
T1-T2 N0 M0	14	(34.1)	NA		NA	
T3-T4 N0 M0	10	(24.4)	NA		NA	
TX N1-N2 MX	11	(26.8)	NA		NA	
TX NX M1	6	(14.6)	NA		NA	
Missing	2		NA		NA	

NA=not applicable.

a Some subjects had several invasive cancers and/or high-risk adenomas.

b Rectum=rectum+recto-sigmoidal junction.

c Distal colon=left colon.

d Proximal colon=right or transverse colon.

e Tumour Node Metastasis (TNM) classification of malignant tumours.

**Table 3:** Comparison of tests sensitivity for advanced neoplasia according to location on the colon

**Location^a^ and type of the most**	**Results of FOBT^b^**			**RSN^c^**
**severe lesion detected**	**G^+^I^+^ (*n*=141)**	**G^+^I^−^ (*n*=249)**	**G^−^I^+^ (*n*=887)**	**2.63 (2.37–2.92)**
*Invasive cancer*	25	2	16	1.48 (1.16–1.89)
Rectum	11	0	12	2.09 (1.36–3.20)
Distal colon	9	2	3	1.09 (0.74–1.60)
Proximal colon	6	0	1	1.17 (0.86–1.58)
*High-risk adenoma*	58	21	204	3.32 (2.70–4.07)
Rectum	11	4	40	3.40 (2.12–5.44)
Distal colon	44	11	122	3.02 (2.38–3.82)
Proximal colon	3	6	42	5.00 (2.55–9.82)
*Small adenoma*	17	40	194	3.70 (2.82–4.87)
Rectum	2	10	44	3.83 (2.08–7.08)
Distal colon	8	18	99	4.12 (2.75–6.15)
Proximal colon	8	13	75	3.95 (2.54–6.14)

a Some subjects had several invasive cancers and/or high-risk adenomas and several locations.

b FOBT=faecal occult blood test; G^+^/G^−^=positive/negative guaiac FOBT; I^+^/I^−^=positive/negative immunochemical FOBT.

c RSN=ratio of sentivities; RSN>1=sensitivity of immunochemical FOBT is greater than that of guaiac FOBT.

**Table 4:** Extension status of colorectal cancer

	**Rectum**				
	**G^+^I^+^^a^**	**G^−^I^+^^a^**	**Total**	**Distal colon**	**Proximal colon**
T1-T2 N0 M0	3	5	8	6	0
T3-T4 N0 M0	4	2	6	3	1
TX N1 MX	2	3	5	4	3
TX NX M1	2	1	3	1	2

a No cancer was detected in subject with a negative I-FOBT.
